# Tundra Type Drives Distinct Trajectories of Functional and Taxonomic Composition of Arctic Fungal Communities in Response to Climate Change – Results From Long-Term Experimental Summer Warming and Increased Snow Depth

**DOI:** 10.3389/fmicb.2021.628746

**Published:** 2021-03-12

**Authors:** József Geml, Luis N. Morgado, Tatiana A. Semenova-Nelsen

**Affiliations:** ^1^MTA-EKE Lendület Environmental Microbiome Research Group, Eszterházy Károly University, Eger, Hungary; ^2^Naturalis Biodiversity Center, Leiden, Netherlands; ^3^Department of Biosciences, University of Oslo, Oslo, Norway

**Keywords:** climate change, fungal ecology, International Tundra Experiment, metabarcoding, tundra

## Abstract

The arctic tundra is undergoing climate-driven changes and there are serious concerns related to the future of arctic biodiversity and altered ecological processes under possible climate change scenarios. Arctic land surface temperatures and precipitation are predicted to increase further, likely causing major transformation in terrestrial ecosystems. As a response to increasing temperatures, shifts in vegetation and soil fungal communities have already been observed. Little is known, however, how long-term experimental warming coupled with increased snow depth influence the trajectories of soil fungal communities in different tundra types. We compared edaphic variables and fungal community composition in experimental plots simulating the expected increase in summer warming and winter snow depth, based on DNA metabarcoding data. Fungal communities in the sampled dry and moist acidic tundra communities differed greatly, with tundra type explaining ca. one-third of compositional variation. Furthermore, dry and moist tundra appear to have different trajectories in response to climate change. Specifically, while both warming and increased snow depth had significant effects on fungal community composition and edaphic variables in dry tundra, the effect of increased snow was greater. However, in moist tundra, fungal communities mainly were affected by summer warming, while increased snow depth had a smaller effect and only on some functional groups. In dry tundra, microorganisms generally are limited by moisture in the summer and extremely low temperatures in winter, which is in agreement with the stronger effect of increased snow depth relative to warming. On the contrary, moist tundra soils generally are saturated with water, remain cold year-round and show relatively small seasonal fluctuations in temperature. The greater observed effect of warming on fungi in moist tundra may be explained by the narrower temperature optimum compared to those in dry tundra.

## Introduction

The arctic tundra is being transformed by a wide range of climate-driven processes and there are serious concerns related to the future of arctic biodiversity because of the threats represented by climate change (Wookey, 2007). In addition to the profound consequences for arctic biota, nutrient cycling in the Arctic is of paramount importance for global change (Tarnocai et al., 2009). The arctic tundra occupies an area of 8 million km^2^ and stores a great portion of the Earth’s soil carbon (C) that is critically important in global C cycles and climate feedback (Callaghan et al., 2004; Tarnocai et al., 2009).

The arctic tundra is considered a maritime biome, because of approximately 80% of its area being located within 100 km of a coastline (Walker et al., 2005) and because arctic sea surface and sea ice cover temperature have been shown to be closely linked to adjacent land surface temperature, precipitation, and primary productivity (Bhatt et al., 2010). Due to retreating sea ice, arctic land surface temperatures have increased and will continue to increase, causing major changes in terrestrial ecosystems (Kaufman et al., 2009). Furthermore, pronounced increase in arctic precipitation is predicted due to increased local surface evaporation of the Arctic Ocean, as well as greater moisture inflow from lower latitudes (Kattsov and Walsh, 2000; Stocker et al., 2013; Bintaja and Selten, 2014). Because most of the precipitation falls as snow, deeper snow is expected in many parts of the Arctic (Bintaja and Selten, 2014).

Warming-induced changes have already been observed in terrestrial arctic ecosystems, including higher microbial activity and resulting increased plant nitrogen (N) availability (Chapin, 1983; Aerts, 2006), faster C turnover in soils (Hobbie and Chapin, 1998; Shaver et al., 2006), and compositional shifts in land surface vegetation (Chapin et al., 1995; Bret-Harte et al., 2002). For example, shrub cover and abundance has increased, which is expected to have positive feedbacks on ecosystem change and greater climate forcing (Sturm et al., 2001). Greater shrub size leads to increased local accumulation of snow in winter, resulting in increased soil insulation, higher winter and spring soil temperatures, and higher rates nutrient mineralization, with positive feedback on shrub growth and expansion (Sturm et al., 2005). Moreover, the expansion of shrubs lowers surface albedo and increases regional summer temperatures, providing another positive feedback to warming (Chapin et al., 2005).

Fungi play key functional roles in terrestrial arctic ecosystems as mutualistic symbionts, pathogens, and decomposers. The vast majority of arctic plants are highly dependent on associations with mycorrhizal fungi for survival in these nutrient-limited environments (Hobbie et al., 2009; Bjorbækmo et al., 2010). Moreover, non-mycorrhizal root endophytic fungi are ubiquitous in arctic-alpine plants (Newsham et al., 2009), their diversity, identity, and ecological roles are scarcely known. Because many fungi of various functional guilds directly interact with plants in a variety of manners, fungi are expected to play important roles in climate-driven changes in arctic vegetation.

Soil fungi are known to respond strongly to warming and elevated nutrient levels (Clemmensen et al., 2006). While vascular plant species diversity is strongly influenced by summer temperatures, soil temperature may be important as a determining factor of soil fungal diversity, particularly in winter, when most fungal metabolic activity takes place (Nemergut et al., 2005). As such, snow depth (the thickness of the insulating layer) and its spatial distribution are expected influence soil fungal communities at small spatial scales. In this study, we analyzed DNA metabarcoding data generated from long-term (18 years) experimental plots simulating climate change in the Alaska Arctic. We compared community composition of soil-borne fungi across sampling plots with (1) ambient and experimentally increased summer air and near-surface soil temperature; (2) ambient and experimentally increased snow depth; and with (3) combined treatment of increased summer warming and increased snow depth in dry heath and moist tussock tundra. We aimed to answer (1) how composition of fungal functional groups change in response to long-term increase in winter snow depth, summer temperature, and their combination; and (2) whether there are differences in responses between dry and moist tundra.

## Materials and Methods

### Study Site and Experimental Design

The sampling was conducted at the International Tundra Experiment (ITEX) long-term research site in the Toolik Lake region in the northern foothills of the Brooks Range, AK, United States (68°37'N, 149°32'W; 760 m above sea level; Walker et al., 1999; Welker et al., 2000; Pattison and Welker, 2014). The region belongs to the bioclimatic subzone E that is the warmest subzone of the arctic tundra, where mean July temperatures ranges from 9 to 12°C and annual precipitation ranges from 200 to 400 mm, of which ca. 50% falls as snow (Walker et al., 2005). The two main vegetation types of the sampled site are dry acidic heath tundra, characterized by *Dryas octopetala*, *Salix polaris*, *Vaccinium* species, and fruticose lichens, and moist acidic tussock tundra, dominated by *Betula nana*, *Salix pulchra* species, the sedge *Eriophorum vaginatum*, and several peat moss species (*Sphagnum* spp.). Detailed descriptions of the plant communities can be found in Walker et al. (1999).

As a part of ITEX network, hexagonal open top chambers (OTCs) and the snow fences were established in 1994, in both the dry (D) and moist (M) tundra, to increase summer air and upper soil temperature and winter snow depth, respectively (Welker et al., 2000). The OTCs, made of translucent fiberglass, have a 1 m^2^ surface area and they increase the summer air and upper soil temperature by a mean daily average of 1.5–2°C measured at 15 cm height and 5 cm depth, respectively (Marion et al., 1997; Jones et al., 1998; Walker et al., 1999). Since 1994, the OTCs were set up every year when 50% of the ground area of the plot became snow-free (late May or early June) and were removed at the end of August or early September, based on the ITEX protocol (Welker et al., 1997). Snow fences, 2.8 m tall and 60 m long, were erected to create a ca. 60-m leeward snow drift with multiple snow depth zones. The soil sampling was focused on the intermediate zone near the center of the experimental setup, corresponding to ca. 1–1.5 m winter snow depth. The average winter soil temperatures 2 cm below the surface were −2.9°C (±0.2) and −4.7°C (±0.2) in the increased snow depth plots and in the control plots with ambient snow depth, respectively (Pattison and Welker, 2014). The lowest soil temperatures recorded for the snow fence plots were ca −7°C vs. ca. −35°C observed for the control plots (Walker et al., 1999; Schimel et al., 2004). Changes in abiotic factors resulting from the increased snow depth have led to marked increases in aboveground plant biomass as well as compositional shifts in vegetation, as described in detail in Walker et al. (1999, 2006), Wahren et al. (2005), Welker et al. (2005), Mercado-Diaz (2011), and Pattison and Welker (2014).

### Data Generation

Soil sampling was done in July, 2012, as described by Morgado et al. (2015, 2016) and Semenova et al. (2015, 2016). In each tundra type, we sampled five replicate plots in the summer warming (W) and increased snow depth (S) treatments, their combination (SW) as well as in the control (C) plots, located adjacent to the experimental treatments. In each replicate plot, five soil cores of 2 cm diameter and 20 cm depth were taken. Both organic and mineral layers were included in the soil cores, while coarse litter, moss, gravel, and coarse roots were removed. Soil cores from each replicate plot were thoroughly mixed and kept frozen until lyophilization. In total, 200 soil cores across 40 plots were sampled.

We extracted DNA from soil samples using Macherey-Nagel NucleoSpin Soil kit (Macherey-Nagel Gmbh and Co., Düren, Germany) with SL2 lysis buffer, which is more suitable for soils rich in organic matter. We used approximately 0.5 ml (ca. 0.2–0.5 g) of soil for DNA extraction and the volume of elution buffer was set to 30 μl. DNA extraction was carried out twice for each sample. The remaining parts of the lyophilized samples were used for soil chemical analyses to measure pH, electrical conductivity (EC) following protocols described in Sparks et al. (1996), and total C and N contents, using vario MAX cube CNS analyzer (Elementar Analysensysteme GmbH, Germany) based on the manufacturer’s protocol.

The PCR and sequencing protocols were as in Geml et al. (2015, 2016), described here briefly. Forward primer fITS7 (Ihrmark et al., 2012) and reverse sample-specific primer ITS4 (White et al., 1990) were used to amplify the ITS2 rDNA region (ca. 250 bp). The ITS4 primer was labeled with sample-specific Multiplex Identification DNA-tags. We used the following PCR protocol for all samples and for the positive and negative controls: one cycle of 95°C for 5 min, and then 25 cycles of 95°C for 20 s, 56°C for 30 s, and 72°C for 1.5 min, ending with one cycle of 72°C for 7 min. DNA concentrations of the PCR products were checked using QIAxcel Advanced System (QIAGEN). Emulsion PCR and Ion Torrent sequencing was carried out at the Naturalis Biodiversity Center with Ion 318TM Chip. The PGM was programmed to split the obtained reads in 40 files, corresponding to the samples, according to MID tags attached to the reverse primer.

### Bioinformatic Analyses

The initial clean-up of the raw sequence data was carried out using the online platform Galaxy,^1^ in which the sequences were sorted according to samples, and adapters (identification tags) and primers were removed. The poor-quality ends were trimmed off with Geneious Pro 5.6.1 (BioMatters, New Zealand), based on 0.02 error probability limit. Quality filtering was done using USEARCH v.8.0 (Edgar, 2010) by truncating all sequences to 200 bp and discarding sequences with expected error > 1. High-quality sequences that passed quality filtering were grouped into operational taxonomic units (OTUs) at 97% sequence similarity with USEARCH, with putatively chimeric sequences discarded. Pairwise similarity searches against the latest USEARCH/UTAX version (8.2) of the curated UNITE fungal ITS sequence database containing identified fungal sequences (Kõljalg et al., 2013) were used for taxonomic classification and to assign OTUs groups to Species Hypothesis groups. We excluded global singletons and OTUs with <80% similarity to a fungal sequence in UNITE. The resulting final dataset contained 4,405 OTUs and 1,941,539 high-quality sequences. We assigned OTUs to functional groups following Tedersoo et al. (2014), supplemented by information regarding the isolation source of curated reference sequences in UNITE. This Targeted Locus Study project has been deposited at DDBJ/EMBL/GenBank under the accession KEOG00000000. The version described in this paper is the first version, KEOG01000000.

### Statistical Analyses

Unless otherwise noted, all statistical analyses were done in R (R Core Team, 2013). The fungal community matrix was normalized (rarefied) by random subsampling to the smallest library size (16,482 reads) on a per-sample basis using the *vegan* package (Oksanen et al., 2012). We excluded OTUs that were found in only one sample, resulting in a matrix containing 2,520 OTUs present in at least two samples, which was the basis for further analyses.

We statistically compared OTU richness and relative abundance of fungal functional groups among the samples with ANOVA and Tukey’s HSD test and presented these graphically as boxplots with *ggplot2* (Wickham, 2016). Compositional differences among samples were visualized using non-metric multidimensional scaling (NMDS) in *vegan* with Bray-Curtis distance measure on the Hellinger-transformed matrix. We performed PerMANOVA (adonis) with 9,999 permutations to estimate the amount of variation explained by the tundra type and the experimental manipulations of summer warming and increased snow depth in a combined model. The combined warming and increased snow depth treatments were assigned to both experimental manipulation types. The distribution of fungal OTUs among tundra types and treatments was visualized in a network with *sna* (Butts, 2008).

## Results

### Patterns of Fungal Richness and Abundance and Edaphic Variables

**Figure 1:**
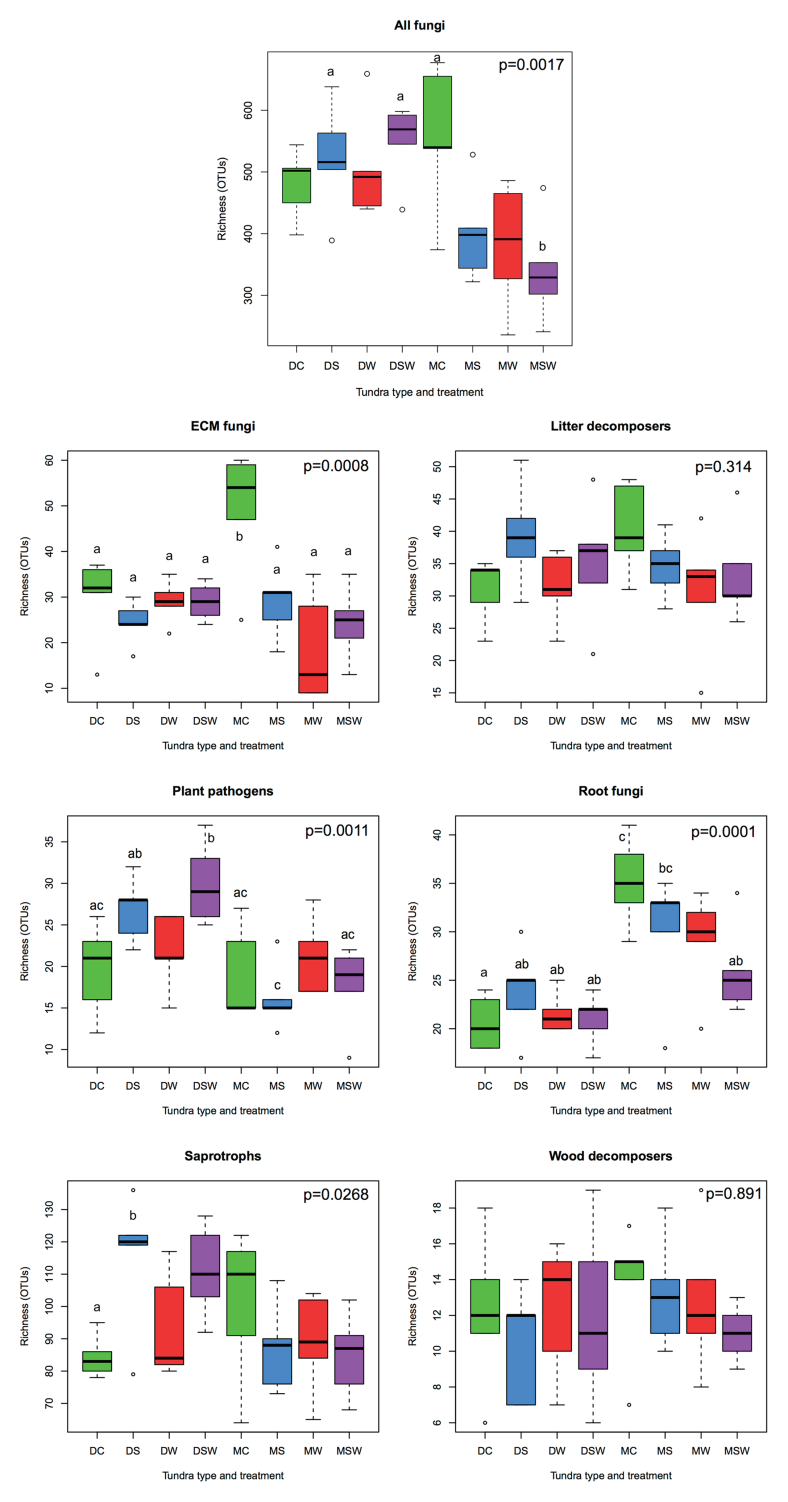
Comparisons of richness of functional groups of arctic fungi across dry and moist tundra and experimental treatments. Means were compared using ANOVA and Tukey’s HSD tests, with letters denote significant differences. D, dry tundra; M, moist tundra; W, summer warming; S, increased winter snow depth; WS, combined summer warming and increased snow depth treatments.

We detected 484 fungal genera among the matching taxa, of which 320 belonged to Ascomycota, 158 to Basidiomycota, and the rest to more early-diverging fungal lineages. Because the taxonomic groups present at the sampling sites have been discussed in detail by Morgado et al. (2015, 2016) and Semenova et al. (2015, 2016), we only present a brief list of the most diverse genera in the complete dataset. The five most OTU-rich genera included *Cladophialophora* (53 OTUs), *Archaeorhizomyces* (36), *Pezoloma* (34), *Meliniomyces* (30) and *Hyaloscypha* (27) in Ascomycota and *Serendipita* (80), *Cortinarius* (69), *Tomentella* (53), *Inocybe* (43), and *Mycena* (29) in Basidiomycota. Of the non-Dikarya genera, Mortierella (47) was by far the most diverse. Total fungal richness was similar in all dry tundra sites, with a non-significant increase in S and SW treatments relative to the control, while all experimental manipulations resulted in a clear decrease in fungal richness in the moist tundra, although only the combined SW treatment was statistically different from the control (Figure 1). With respect to functionality, 1,186 of 2,520 OTUs present in at least two samples were assigned to functional groups. Significant differences in richness among treatments were observed in four of the six functional groups analyzed (Figure 1). Richness of ectomycorrhizal (ECM) fungi was significantly higher in control than any of the treatment plots in moist tundra. The second highest richness was found in the dry tundra control plots, but it was not significantly different from any of the experimental treatments. In plant pathogenic fungi, highest richness was observed in the S and SW treatments, although only the latter differed significantly from the control. No statistical differences were observed in the moist tundra for the same functional group. Conversely, root-associated non-mycorrhizal fungi showed highest richness in moist control plots and decreased in all treatment plots, with significant difference observed between control and the SW and S treatments, while richness values were uniformly low for all treatments in the dry tundra. Saprotrophic fungal richness was highest in the dry S treatment, with no statistical differences among the other treatments (Figure 1).

**Figure 2:**
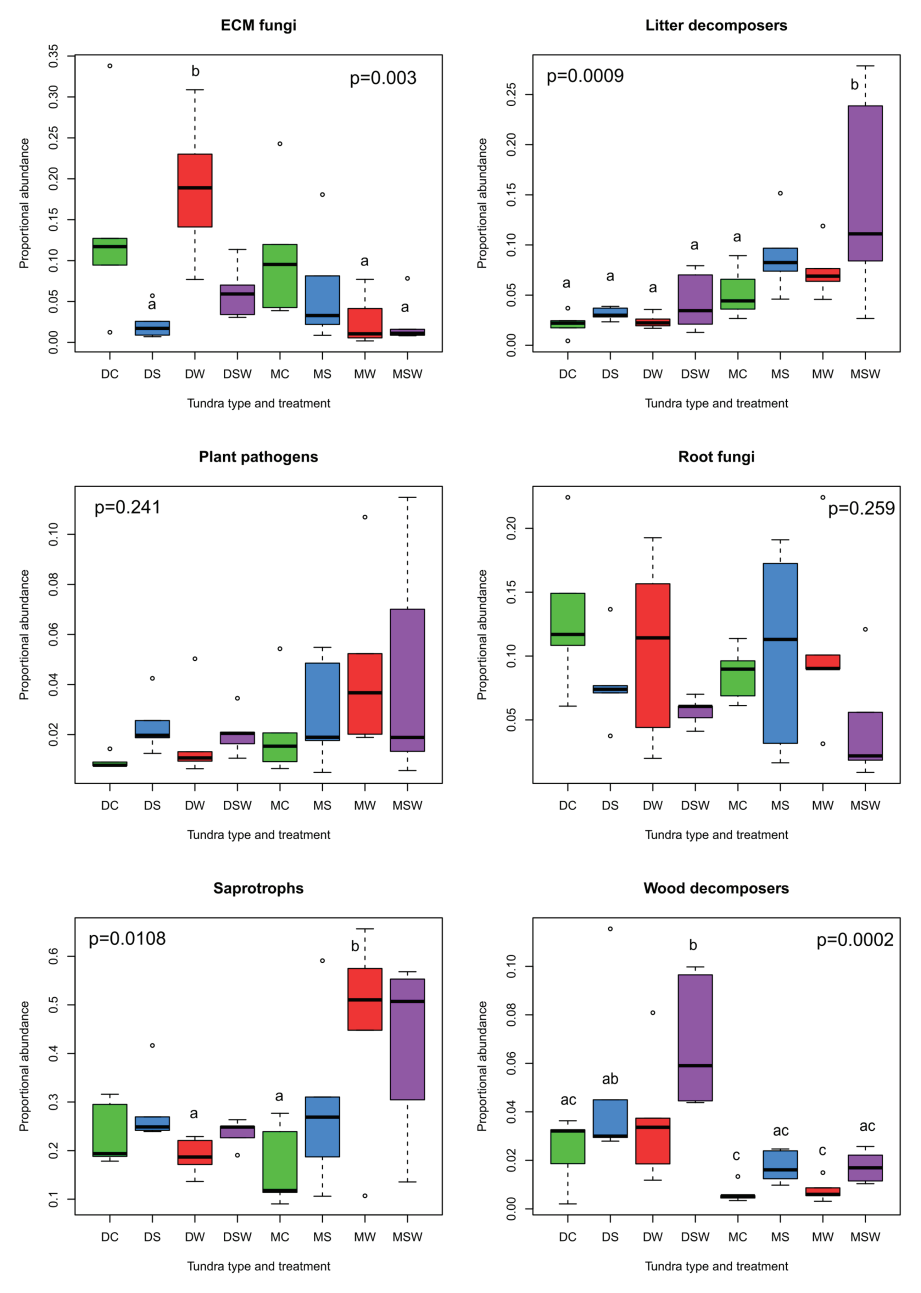
Comparisons of proportional abundance of functional groups of arctic fungi across dry and moist tundra and experimental treatments. Means were compared using ANOVA and Tukey’s HSD tests, with letters denote significant differences. D, dry tundra; M, moist tundra; W, summer warming; S, increased winter snow depth; WS, combined summer warming and increased snow depth treatments.

With respect to proportional abundance, four out of six functional groups exhibited significant differences, with the important differences that although litter and wood decomposers did not differ in proportional abundance among tundra types and treatments, they showed the strongest changes in proportional abundance (Figure 2). The abundance of litter fungi consistently increased in the snow addition plots in dry and moist tundra, with the strongest increase observed in the latter, particularly in the SW treatment. Conversely, experimental manipulations seemed to have stronger effects on wood decomposers in the dry tundra, with a significant increase in richness observed in the SW treatment relative to the control. Even though S and SW treatments resulted in higher observed richness in the moist tundra as well, the difference from the control was not significant. In ECM fungi, the only significant differences were observed between the high value of proportional abundance observed in the dry W and the low values in the dry S and moist W and SW treatments. In generalist saprotrophs, the moist W and SW showed the highest proportional abundance, with the W being statistically different from the control (Figure 2).

**Figure 3:**
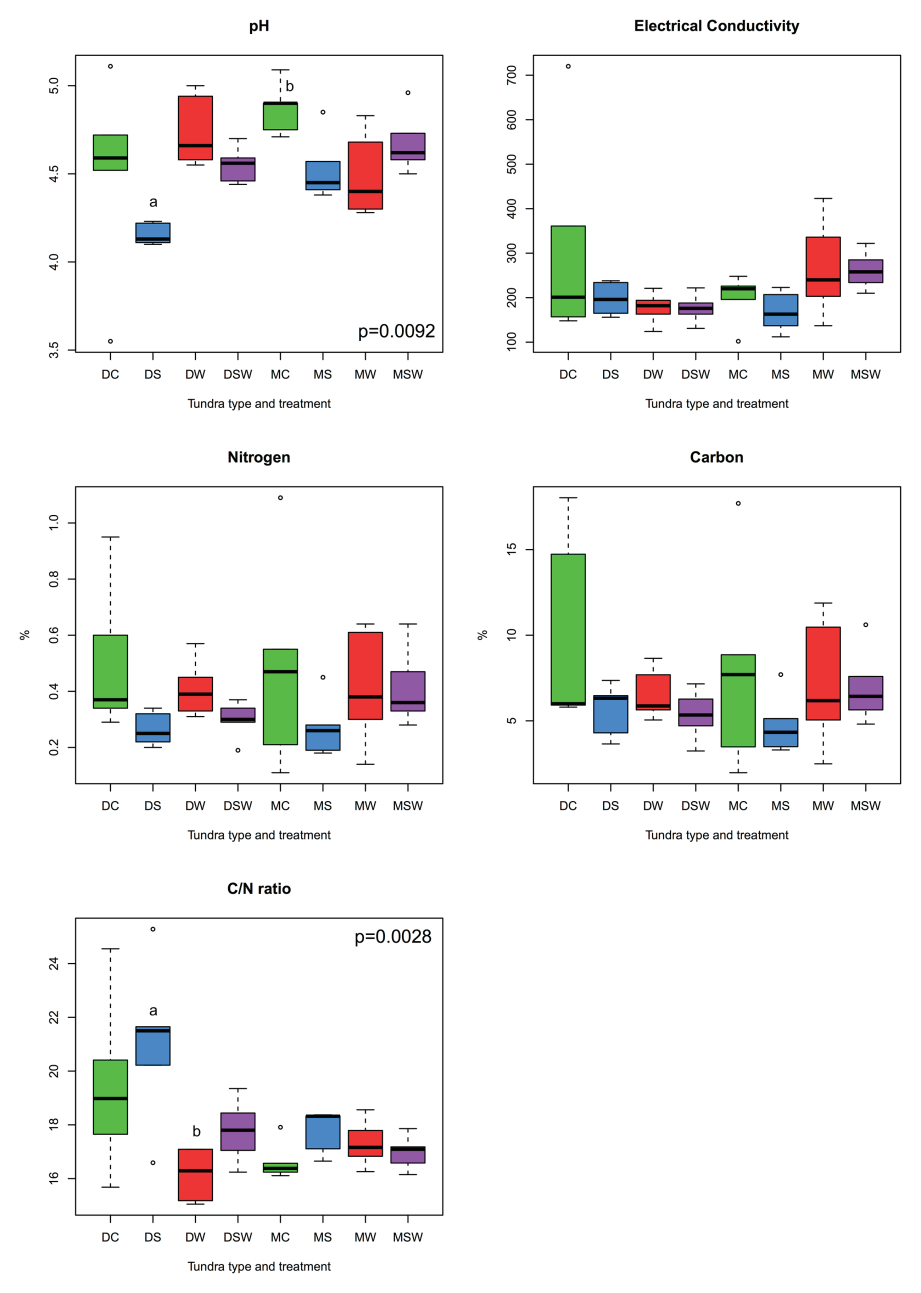
Comparisons of measured edaphic variables across dry and moist tundra and experimental treatments. Means were compared using ANOVA and Tukey’s HSD tests, with letters denote significant differences. D, dry tundra; M, moist tundra; W, summer warming; S, increased winter snow depth; WS, combined summer warming and increased snow depth treatments.

Of the five edaphic variables tested (pH, EC, C and N content, and C/N ratio), only pH and C/N ratio differed among the experimental treatments (Figure 3). In both tundra types, soil pH was lower under increased snow depth than in the control, although the difference mostly remained non-significant. The pattern of C/N ratio was practically opposite of the trend observed for pH. These changes were greater in the dry tundra than in the moist, primarily under increased snow depth in the dry tundra, that had the lowest pH and the highest C/N ratio values among all treatments.

### Comparing Fungal Community Composition Among Tundra Types and Treatments

PerMANOVA revealed that tundra type was by far the strongest driver for the structuring of fungal communities, explaining between 21 and 42% of the variation in community composition of the various functional groups when considering all sites (Supplementary Table S1). Therefore, additional PerMANOVA analyses were carried out for all functional groups to evaluate the effects of treatment on compositional variation separately in the dry and moist tundra. In dry tundra, the effects of experimental increase in snow depth, soil pH, and EC, and to a smaller extent warming and soil N content, tended to explain the greatest variation in community structure, but substantial differences were observed among the functional groups (Supplementary Table S1). Wood decomposer fungi differed most from the above general trend: C/N ratio was the most influential to their community composition, followed by pH and warming treatment, with increased snow depth having non-significant contribution to explained variance. In moist tundra, the contribution of warming was significant for most functional groups, except ECM fungi and wood decomposers. The effect of increased snow depth generally was weaker, with significant treatment effect on community composition being observed only in ECM and other root-associated fungi in the moist tundra. Of the edaphic variables, EC explained surprisingly large fractions of the variation in community composition in ECM, litter decomposer and generalist saprotrophic fungi (all above 10%).The effect of soil pH was only significant in ECM and other root-associated fungi.

**Figure 4:**
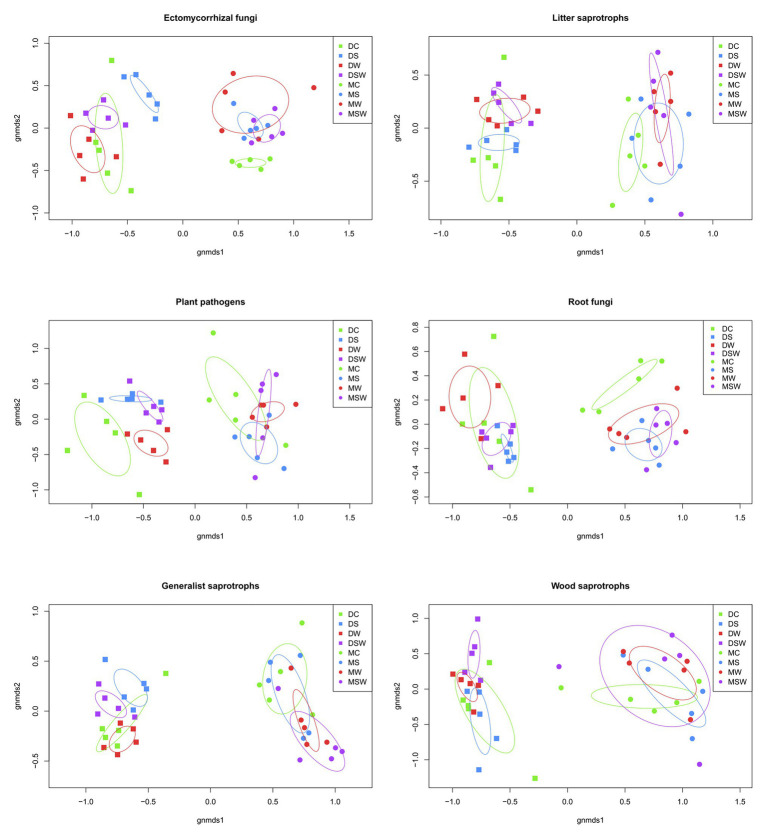
Non-metric multidimensional scaling (NMDS) ordination plots of the six most diverse functional groups of arctic fungi based on Hellinger-transformed data. For each treatment, circles indicate the SD. D, dry tundra; M, moist tundra; W, summer warming; S, increased winter snow depth; WS, combined summer warming and increased snow depth treatments.

**Figure 5:**
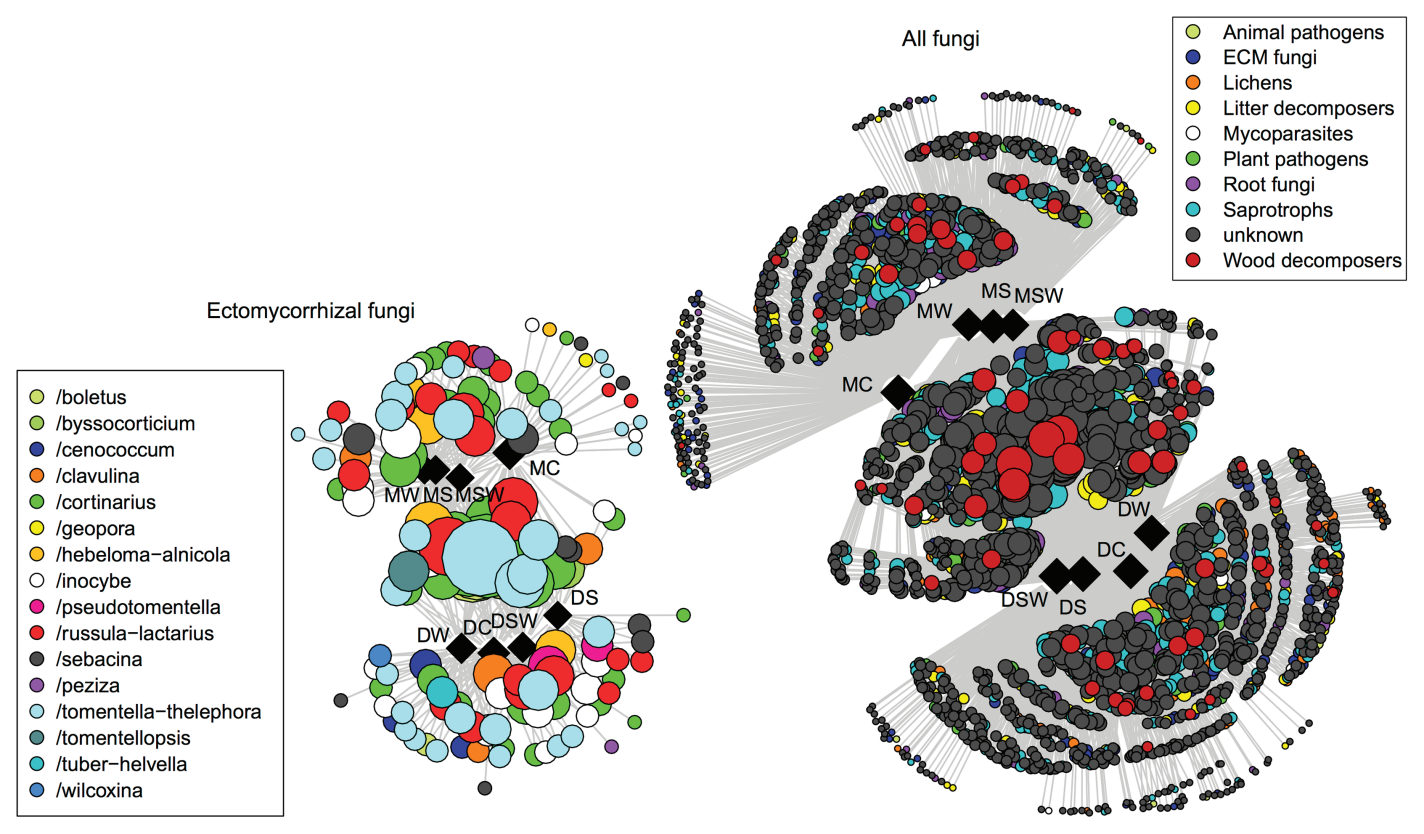
Distribution of operational taxonomic units (OTUs) of ectomycorrhizal and all fungi as visualized by network analysis for the different tundra types and experimental treatments. Squares and circles indicate sample categories and OTUs, respectively. Circles representing OTUs are connected with straight lines to samples (squares) they occur in. Centrally located OTUs are shared among multiple treatments and/or tundra types. Circle size is proportional to the number of samples (in respective habitats and/or treatments) the OTU is present in.

Non-metric multidimensional scaling plots of the selected functional groups of fungi confirmed the dominant contribution of tundra type to community structure as detailed above (Figure 4). However, network graphs of all fungi showed that a substantial proportion of OTUs from different functional groups were shared between dry and moist tundra and among treatments (Figure 5). In addition, each treatment contained unique OTUs, particularly the control plots contained many OTUs that were absent from the treatment plots. The network of ECM fungal OTUs showed that the /tomentella-thelephora, /cortinarius, and /russula-lactarius lineages dominated the ECM community. Although these lineages had many OTUs restricted to either dry or moist tundra, OTUs that occurred in both tundra types came overwhelmingly from these lineages. OTUs in the/inocybe lineage, which is among the most diverse and widespread lineages of arctic fungi (Geml et al., 2012b), appeared to be more restricted to a certain tundra type (Figure 5).

## Discussion

This study provides insights into the compositional dynamics of arctic fungi, highlighting their diversity, abundance, and distribution in dry and moist acidic tundra and their possible responses to warming and increased winter precipitation. The deep sequence data presented here, coupled with the measured edaphic variables, clearly show that (1) arctic fungal community composition at the selected sites is determined principally by tundra type; (2) experimental warming and increased snow depth have different effects edaphic variables and fungal communities on dry vs. moist tundra; (3) a substantial proportion of the effects of summer warming and increased winter snow depth on fungal communities likely is mediated through changes in edaphic conditions, and that (4) there are important differences among functional groups of fungi in how they respond to increases in summer temperatures, snow depth, and related alterations in soil chemistry.

Despite the spatial proximity of the dry and moist tundra plots (<500 m), the overwhelming compositional differences in fungi suggest that many fungi appear to be sensitive to the differences in hydrology and vegetation between these tundra types. However, it is worth to note that a relatively large fraction of the fungal community is shared between the dry and moist tundra and among various treatments. It is plausible that climate change and related changes in abiotic factors will favor these fungi with apparently wide niche breadth as opposed to those with narrower observed niche breadth that are restricted to a certain habitat, particularly to the control sites. Because dispersal generally is not considered a limiting factor in arctic fungi (Geml, 2011; Geml et al., 2012a,b), it is presumed that, other than stochastic factors related to “founder effects,” environmental filtering drives fungal community composition in the sampling area. Landscape-scale studies on the compositional dynamics of arctic fungi among several tundra habitats spanning moisture and acidity gradients are needed to learn more about fungal niches in the Arctic.

In addition to the differences in fungal species pools in dry and moist acidic tundra at the sampling sites, there were also distinct responses of the dry and moist tundra fungal communities to experimental warming and increase in snow depth. This suggests that species pool and pre-disturbance environmental factors, such as site hydrology and resulting differences in vegetation structure, likely drive response trajectories of fungal communities, similar to what has been reported for plants as well (Walker et al., 2006; Mercado-Diaz, 2011; Pattison and Welker, 2014). For example, the strong responses in fungal communities to warming in the moist tundra are in agreement with more pronounced plant community responses in the moist than in the dry tundra, although it is unclear to what extent compositional shifts in fungal communities drive vegetation shifts or are driven by them. In addition, differential fungal responses to warming in the dry and moist tundra likely are related to differences in natural temperature fluctuations in the dry and moist tundra. Moist tundra soils generally experience less temperature variation due to higher water content and a dense peat moss layer that buffers changes in air and surface temperature. In dry tundra, where vegetation cover generally is below 50% and soils are relatively dry, the ca. 2°C warming during the summer may represent a negligible change relative to the high variation in temperatures experienced under normal circumstances. With respect to increased snow depth, it is important to note that greater snow depth increases winter soil temperature as well as soil moisture, particularly in early summer, and these effects cannot be decoupled. The strong effect of available moisture on fungal growth has repeatedly been shown in dry tundra (Jones et al., 1998; Schimel et al., 2004; Wahren et al., 2005; Mercado-Diaz, 2011). Therefore, the stronger response by the community of the dry heath tundra may partly be explained by partially alleviating water stress in this habitat. In addition, the edaphic data presented here indicate that of all treatments, S treatment plots in the dry tundra differ most from the rest in soil pH and C/N ratio. More precisely, these plots have the most acidic soils that contain the most decay-resistant organic matter. The fact that the richness of generalist saprotrophs was the highest in this treatment, but not their relative abundance, may indicate that the low nutrient levels favor a high diversity of relatively slow-growing saprotrophic fungi. Moist tundra soils are mostly saturated by water throughout the growing season and winter snow cover is deeper and more homogenous than in the dry tundra community, where soils tend to have little or no snow cover and low moisture content, resulting in very cold temperatures and frequent desiccations. It is noteworthy that, particularly in the dry tundra, the compositional effects of the combined SW treatment resulted in communities that appeared to be intermediate between those of S or W only, as suggested by the NMDS results. Similarly, opposite trends in S and W treatment were also observed to some extent in richness and in soil pH and C/N ratio, though they remained mostly non-significant. This suggests that in the dry tundra, these S and W treatments may have divergent effects on the fungal communities, possibly because they alter soil moisture and soil chemical processes in opposite directions.

ECM and non-mycorrhizal root fungi appear to be most diverse in the moist tundra, but their high richness is not coupled with high overall abundance. Their decreasing trends in richness and abundance and the strong compositional shifts in the moist tundra treatment plots, suggest that the altered abiotic conditions, and possibly the resulting differences in vegetation, are no longer suitable for many root-associated fungi that normally inhabit moist acidic tussock tundra. This is surprising, because ECM fungal richness and abundance generally correlate with host plant abundance (Tedersoo et al., 2014) and higher density and biomass of ECM shrubs were reported in increased snow depth plots in both tundra types (Mercado-Diaz, 2011; Pattison and Welker, 2014). Differences in nutrient scavenging capabilities among ECM fungal species under the altered conditions may explain some of the observed patterns, as changes in plant community and N dynamics had been reported to be more strongly affected by the increased snow depth in the moist than in the dry tundra (Schimel et al., 2004; Wahren et al., 2005; Mercado-Diaz, 2011). For example, presenting data from the S and W plots analyzed here, Morgado et al. (2015, 2016) argued that ECM fungi with exploration types adapted to labile N uptake generally showed decreasing richness under increased snow depth, while the richness of ECM fungi with exploration types adapted to acquire recalcitrant soil N were not affected by increased snow depth. Although Morgado et al. (2015, 2016) did not analyze data from the combined SW plots that are presented here, their observations, coupled with the higher C/N values observed here suggest that the capability of acquiring recalcitrant N may represent an important environmental filter for ECM fungi under increased snow depth and appear to be explain partly the lower richness and abundance of ECM fungi. Alternatively, it could partly be caused by the water-logged, likely anaerobic conditions in the SW plots in the summer (József Geml, pers. obs.). The findings presented here show that the above differences likely are at fine taxonomic scales, i.e., at species level, as most ECM genera included numerous OTUs that were specific to a habitat and others that were shared among tundra types and treatments, indicating that in most genera comprise species that represent a wide spectrum of ecological preferences and niche breadths. It is well-known that ECM fungi compete with saprotrophic fungi for water and nutrients (Orwin et al., 2011) and the decline in ECM fungi, the increased abundance of decomposers, and higher rates of decomposition with increasing summer and winter soil temperatures (as discussed below), may result in decreased C sinks in tundra soils, as plants in cold climates are known to transfer up to 50–70% of their C uptake directly to ECM fungal mycelia (Clemmensen et al., 2013).

The increased relative abundance of decomposers of litter and wood in the snow addition treatments (S and SW) in moist and dry tundra, respectively, may be related to the abilities of these fungi to take advantage of more available substrate as well as more favorable conditions for enzymatic degradation. Increased quantities of litter and woody debris resulting from the greater above-ground biomass of shrubs and graminoids have been reported for these plots with increased snow depths as well as with summer warming (Hollister et al., 2005; Wahren et al., 2005; Mercado-Diaz, 2011; Semenova et al., 2015) and microbial decomposition rates have been found to be greater (Sistla et al., 2013). Saprotrophic fungal activity generally is correlated with decomposition rates and CO_2_ flux between the terrestrial and atmospheric pools. Therefore, an increased abundance of saprotrophic fungi may increase fungal enzymatic activity in soils, with higher rates of C mineralization and greater CO_2_ emission to the atmosphere (Guhr et al., 2015). The greater aboveground plant biomass of the moist tundra treatment plots also is in agreement with the higher proportional abundance of plant pathogenic fungi that could benefit from greater host biomass. Greater snow depth increases winter soil temperature, which is expected to have positive effects on microbial decomposition. The increased abundance of generalist saprotrophs in the increased snow depth and warming plots provides further support for this idea. Overall, the observed changes in richness and abundance decomposer and other saptrophic fungi provides proof for the conceptual framework that increased snow depth provides greater soil insulation, resulting in higher winter and spring-time soil temperatures, and increased rates of nutrient mineralization. This, in turn, favors shrub growth and expansion, which leads to decreased albedo, increased snow trapping, and enhanced CO_2_ release to the atmosphere, providing positive feedback to climate change.

## Conclusion

Fungi play numerous ecological roles of key importance and are involved in a complex array of interactions with other organisms that are still poorly known. We observed greater treatment effect on edaphic variables in dry tundra than in the moist that suggest that dry tundra may be more responsive to climate change, particularly increased winter snow depth. Climate-driven fungal community dynamics and changes in richness and abundance of functional guilds will likely have a wide range of consequences. While the currently prevailing view is that altered plant community composition drives fungal community change in the Arctic, it seems that fungal community composition may change more rapidly and independently of plant communities and that fungi may be particularly well-suited to monitor early responses to environmental changes. It is possible that, in response to experimental manipulations simulating predicted changes in climate, functional groups fungi in our dataset declined or increased under due to alterations in communities of other organismal groups which were not investigated here and with which they could have mutualistic or competitive interactions. Because of the scarcity of information on other components of soil biodiversity in the Arctic in general and at the sampling site in particular, it is difficult to assess the extent to which fungal responses to the above-mentioned manipulations are the results of abiotic factors vs. biotic interactions. Future studies on inter-kingdom interactions, e.g., among fungi, bacteria, myxomycetes and soil invertebrates, are needed to gain a more complete understanding of soil biota and its resilience to climate change.

## Data Availability Statement

The datasets presented in this study can be found in online repositories. The names of the repository/repositories and accession number(s) can be found at: https://www.ncbi.nlm.nih.gov/genbank/, KEOG01000000.

## Author Contributions

JG designed the research and selected sampling sites. JG, LM, and TS-N performed the fieldwork, labwork, and the initial bioinformatics. JG completed the bioinformatics and the statistical analyses, to which LM contributed R scripts. JG wrote the first draft of the paper and all authors contributed to the revisions of the manuscript that resulted in the first submitted version.

### Conflict of Interest

The authors declare that the research was conducted in the absence of any commercial or financial relationships that could be construed as a potential conflict of interest.

## Footnotes

^1^https://main.g2.bx.psu.edu/root
