# Supplementary material for: Tundra Type Drives Distinct Trajectories of Functional and Taxonomic Composition of Arctic Fungal Communities in Response to Climate Change – Results From Long-Term Experimental Summer Warming and Increased Snow Depth
Source: Front Microbiol. 2021 Mar 12;12:628746. doi: 10.3389/fmicb.2021.628746 (PMC7994276; doi:10.3389/fmicb.2021.628746)
Supplement: Supplementary file 1 [file Table_1.doc]

Table 1. Proportion of variation in fungal community composition explained by tundra type, experimental warming and increased snow depth calculated independently with permutational multivariate analysis of variance, based on Hellinger-transformed fungal community matrix. Significant results are in bold. Climatic and edaphic variables that remained significant in the final composite model for each fungal group are shown with an asterisk (*).

| **All plots** | **All fungi** | **ECM fungi** | **Litter decomp.** | **Plant path.** | **Root fungi** | **Saptrotrophs** | **Wood decomp.** |
| --- | --- | --- | --- | --- | --- | --- | --- |
| Tundra type | **31.89*** | **21.17*** | **27.02*** | **24.92*** | **41.09*** | **34.29*** | **31.69*** |
| Warming | 4.03 | 3.98 | **5.88*** | 4.22 | 2.55 | 4.33 | 3.74 |
| Snow depth | 3.74 | **4.23*** | 3.42 | 3.61 | 3.99 | 4.23 | 2.37 |
| pH | **5.87*** | **6.01*** | **5.33** | **7.62*** | **6.35** | **5.56** | 5.08 |
| EC | 3.04 | 2.95 | 3.59 | 3.62 | 3.4 | 3.03 | 1.88 |
| N | 3.56 | 2.91 | 4.36 | 3.63 | 3.43 | 4.24 | 2.19 |
| C | 2.77 | 2.62 | 3.55 | 2.97 | 2.61 | 3.27 | 1.65 |
| C/N | **6.84*** | **5.26** | **5.63** | **8.17*** | **6.4*** | **7.38*** | **6.49*** |
| **Dry tundra** | **All fungi** | **ECM fungi** | **Litter decomp.** | **Plant path.** | **Root fungi** | **Saptrotrophs** | **Wood decomp.** |
| Warming | **9.02** | **11.11*** | **11.78*** | **12.54** | 7.51 | **9.96** | **13.72** |
| Snow depth | **13.78*** | **10.53*** | **10.34*** | **19.53*** | **13.61*** | **19.14*** | 7.27 |
| pH | **14.83*** | **12.38*** | **12.13*** | **20.67*** | **18.41*** | **16.99*** | **16.27** |
| EC | 6.97 | **5.84** | **8.17** | 9.79 | 8.76 | 5.67 | 5.09 |
| N | **8.9*** | **7.45*** | **8.89*** | **12.97*** | **14.17*** | **8.61** | 6.44 |
| C | 7.25 | 6.03 | **7.97** | 8.98 | **10.57*** | 6.98 | 6.14 |
| C/N | **13.89*** | **10.81** | **9.4** | **15.05*** | **16.91*** | **17.94*** | **17.2*** |
| **Moist tundra** | **All fungi** | **ECM fungi** | **Litter decomp.** | **Plant path.** | **Root fungi** | **Saptrotrophs** | **Wood decomp.** |
| Warming | **12.76*** | 7.32 | **10.96** | **9.03*** | **10.21*** | **17.05*** | 7.65 |
| Snow depth | 8.09 | **10.61*** | 6.83 | 7.13 | **12.69*** | 6.73 | 5.15 |
| pH | 7.44 | **10.09*** | 6.72 | 6.47 | **10.44*** | 6.04 | 6.46 |
| EC | **12.18*** | **10.47*** | **11.66*** | 7.59 | 7.52 | **17.53*** | 8.47 |
| N | **8.15** | 7.42 | **9.59** | 6.65 | 6.03 | **12.02** | 6.43 |
| C | **8.22** | 7.61 | **9.79** | 6.67 | 5.86 | **12.1** | 6.39 |
| C/N | 6.66 | 5.7 | 5.59 | 5.78 | 6.83 | 8.65 | 5.03 |
